# Testing for genetic association taking into account phenotypic information of relatives

**DOI:** 10.1186/1753-6561-3-s7-s123

**Published:** 2009-12-15

**Authors:** Hae-Won Uh, Henk Jan van der Wijk, Jeanine J Houwing-Duistermaat

**Affiliations:** 1 Department of Medical Statistics and Bioinformatics, Leiden University Medical Center, PO Box 9600, Leiden 2300RC, The Netherlands

## Abstract

We investigated efficient case-control association analysis using family data. The outcome of interest was coronary heart disease. We employed existing and new methods that take into account the correlations among related individuals to obtain the proper type I error rates. The methods considered for autosomal single-nucleotide polymorphisms were: 1) generalized estimating equations-based methods, 2) variance-modified Cochran-Armitage (MCA) trend test incorporating kinship coefficients, and 3) genotypic modified quasi-likelihood score test. Additionally, for X-linked single-nucleotide polymorphisms we proposed a two-degrees-of-freedom test. Performance of these methods was tested using Framingham Heart Study 500 k array data.

## Background

Several single-gene variants associated with coronary heart disease (CHD) using Framingham Heart Study (FHS) 100 k array data were reported previously [1]. Regression models with generalized estimating equations (GEE) [2] as well as family-based association testing using FBAT [3] were used. Both methods do not utilize all family information available. While the FBAT test statistic is based on the use of offspring genotypes conditional on (informative) parental genotypes, the GEE association test uses all individuals with genotype and phenotype data. The latter usually uses an exchangeable working correlation matrix to account for correlation within each sibship. Hence, available parental information is not optimally used.

Our aim is to use family information efficiently. In this paper we study an association between CHD and candidate genes using the binary outcome of CHD directly. The following methods were investigated: 1) a logistic regression model taking into account familial dependence of the observations using GEE, 2) Cochran-Armitage (CA) trend test taking into account the correlations among related individuals when computing the variance, and 3) the extensions of modified quasi-likelihood score (M_QLS_) test [4]. The last methods also use phenotypic information of ungenotyped family members for an optimal weighting scheme, and can be used for sibships as well as for nuclear families. Because the first two methods are genotypic tests, we extended the allelic M_QLS _test to the corresponding genotypic test (gMQLS), assuming a multiplicative model [5].

Unil now, little has been reported on performance of such test statistics for association on the X chromosome [6,7]. Because the X chromosome represents 2.5% of the human genome for males and 5% for females, information coming from the X chromosome cannot be ignored. To identify X-linked markers for susceptibility to a disease, we investigate statistics to test for association on the X chromosome in a related sample using GEE and sex-stratified allelic M_QLS _test.

## Methods

### Study sample

We analyzed Problem 2 of Genetic Analysis Workshop 16 data, using GeneChip^® ^Human Mapping 500 k Array Set provided by the FHS SHARe (SNP Health Association Resource) project. The large pedigrees (*n *= 841) were broken up into nuclear family units (*n *= 1,902). The data consist of 2,878 subjects in the Offspring Cohort (*n *= 2,555) and their parents in the Original Cohort (*n *= 323). A binary outcome variable was created as any event of hard CHD (*n *= 225). The details of data sets created and used are described in Table 1.

**Table 1:** Description of data used for each method

	Offspring Cohort	Original and Offspring Cohort
No. families	1,767	1,902
		
Size of family	1-7	1-7
		
No. genotyped^a^	2,411	2,722
CHD	153	215
Controls	2,258	2,507
		
Total no.^b^	2,555	2,878
CHD	160	225
No CHD	3	70
Population controls^c^	2,392	2,583
		
Sex		
Males	1,167	1,264
Females	1,388	1,614

^a^CA, MCA, and GEE methods used.

^b^gMQLS method used; genotyped and ungenotyped samples included.

^c^As defined in Eq. (1)

### Single-nucleotide polymorphism (SNP) selection

We checked inheritance error. PLINK version 1.02 [8] was used for preprocessing of data with the following inclusion thresholds: minor allele fequency ≥ 0.01, missing rate per person ≤ 0.1, missing rate per SNP ≤ 0.1, and Hardy-Weinberg equilibrium *p *≥ 0.001. For chromosome 8, by ignoring relatedness between subjects, we conducted allelic tests for the preprocessed 22,207 SNPs (from 27,362 of FHS 500 k SNP resource) using PLINK. Then, 121 SNPs were selected using a threshold of allelic *p*-values < 0.005. For chromosome X, 8,020 SNPs (from 9,828) were tested, and using the same threshold 35 SNPs were selected.

### GEE-based and modified CA trend test

One merit of using pedigrees in a case-control study is that cases with affected relatives might have higher expected frequency of associated alleles than cases without affected relatives. For GEE, an exchangeable working correlation matrix was used to account for correlation within each sibship and each family. However, this correlation is prone to misspecification, and subsequent loss of efficiency may be substantial [9].

Under the null hypothesis of no association between genotype and disease, CA trend test is , where *U *is a sum of weighted differences of genotype counts between cases and controls. When subjects are biologically related, we need to account for their correlations by computing the variance of *U*. Slager and Schaid [10] proposed a method in which the variance and covariance terms can be calculated based on identity-by-decent-sharing probabilities. We calculated the covariance using expected identity-by-decent (2 times kinship coefficient); hence, this method is called the modified Cochran-Armitage (MCA) test.

### M_QLS _test and its extensions

Alternatively, we considered M_QLS _test proposed by Thornton and McPeek [4], which is said to be more powerful and more widely applicable. It distinguishes between unaffected controls and controls of unknown phenotype (general population controls), and it also incorporates phenotypic data of relatives with missing genotypes.

Suppose we have *n *+ *m *sampled individuals with phenotypic information. Let *Y *= (*Y*_1_, ..., *Y*_*n*_) denote genotype data of *n *individuals with non-missing genotype, so that *m *individuals have missing genotype. Let Φ be the kinship matrix of the non-missing genotype individuals, and Φ_*N*, *M *_between missing and non-missing genotype individuals. The entries of the matrix are 1 on the diagonal and 2*ϕ*_*ij *_kinship coefficient between the *i*^th ^and *j*^th ^individual off the diagonal. *A*_*N *_and *A*_*M *_are the column of the phenotype of the respectively non-missing and missing genotype individuals. The entry in *A *for the *i*^th ^individual from the *j*^th ^family is

with 0 <*k *< 1 specified to be the population prevalence of the trait. Then, the statistic is given by

where, *α *= *A*_*N *_+ Φ^-1 ^Φ_*N*, *M *_*A*_*M*_, Γ = *α*^*T*^(Φ*A*_*N *_+ Φ_*N*, *M *_*A*_*M*_)- (1^*T *^*α*)^2 ^(1^*T*^Φ^-1^1^*T*^)^-1^,

, , and .

We extended the allelic M_QLS _test to the corresponding genotypic test, gMQLS, assuming multiplicative model using genotypic mean  and the corresponding variance .

For the X-linked SNPs, a simple allele-based test can be constructed by counting alleles, with males contributing a single allele and females two alleles. Because the assumption that the allele frequency does not vary with sex could not be met, we stratified the analysis by sex, and used the allelic M_QLS _test. To combine the results we combined the two chi-squared tests to obtain a two-degrees-of-freedom test (xMQLS).

The analyses using new methods have been conducted using functions written by the authors in R [11].

## Results

### Association study for autosomal SNPs on chromosome 8

**Figure 1:**
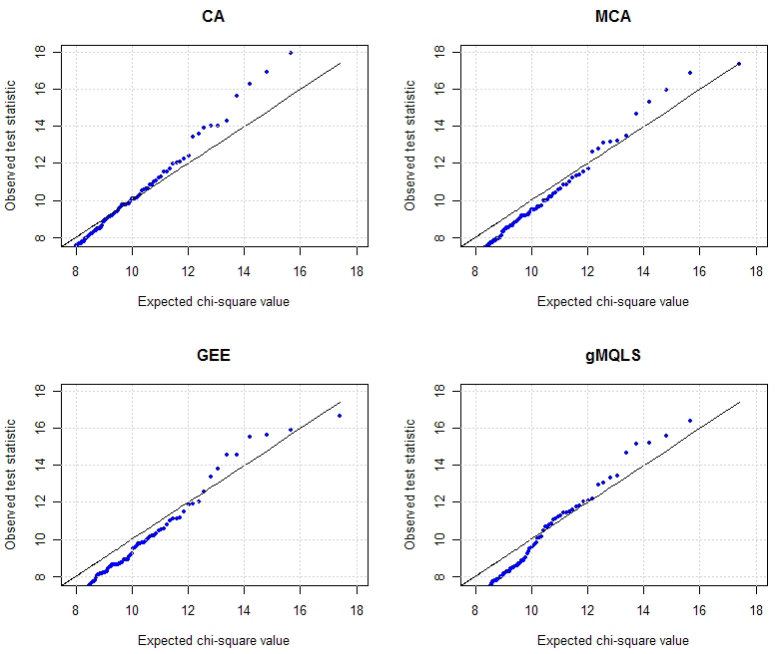
**Quantile-quantile plots of four statistics of 121 SNPs selected on chromosome 8**. For GEE, the statistic  was used.

We compared the following methods: CA, MCA, GEE, and gMQLS. These tests were performed 1) using Offspring Cohort and 2) using the Original and Offspring Cohorts as described in Table 1. Note that for gMQLS, phenotypic information of un-genotyped individuals was also incorporated. The population prevalence of CHD - *k *in Eq. (1) - was set as 5%. To compare type 1 error rates, the quantile-quantile plots of 0.5-percentiles (the percentage of SNPs selected) are depicted in Figure 1. The points below the diagonal indicate that allelic tests ignoring relatedness in PLINK overestimated the association. The results are comparable for these selected SNPs.

In Table 2, the top ten ranking SNPs detected by gMQLS using nuclear families are reported. The gMQLS gave more significant results when information of parental generation was included: for example, the *p*-value decreased from 9.80 × 10^-5 ^to 1.05 × 10^-5 ^for RS17094201. None of the SNPs tested were found to have genome-wide significance (nominal *p *< 5 × 10^-8^).

**Table 2:** *p*-Values of autosomal SNPs on chromosome 8 using (1) Offspring Cohort and (2) Original and Offspring Cohort

	(1) Offspring Cohort				(2) Nuclear family			
		
SNP	CA^a^	MCA	GEE	gMQLS^b^	CA^a^	MCA	GEE	gMQLS^b^
RS17094201	7.76 × 10^-5^	1.27 × 10^-4^	4.40 × 10^-5^	9.80 × 10^-5^	1.16 × 10^-5^	2.22 × 10^-5^	1.23 × 10^-5^	1.05 × 10^-5^
RS12549036	1.90 × 10^-4^	2.96 × 10^-4^	8.62 × 10^-4^	2.65 × 10^-4^	8.75 × 10^-5^	1.48 × 10^-4^	2.15 × 10^-4^	8.46 × 10^-5^
RS4961118	2.33 × 10^-3^	3.15 × 10^-3^	2.86 × 10^-3^	2.10 × 10^-3^	1.67 × 10^-4^	2.71 × 10^-4^	3.80 × 10^-4^	9.93 × 10^-5^
RS6586789	4.92 × 10^-3^	6.38 × 10^-3^	5.54 × 10^-3^	5.01 × 10^-3^	1.47 × 10^-4^	2.40 × 10^-4^	1.91 × 10^-4^	1.23 × 10^-4^
RS16920160	2.27 × 10^-4^	3.49 × 10^-4^	5.70 × 10^-4^	7.94 × 10^-5^	5.93 × 10^-4^	8.89 × 10^-4^	7.63 × 10^-4^	1.63 × 10^-4^
RS3812476	1.80 × 10^-3^	2.47 × 10^-3^	3.16 × 10^-3^	1.02 × 10^-3^	4.42 × 10^-4^	6.75 × 10^-4^	7.14 × 10^-4^	1.90 × 10^-4^
RS11989122	4.15 × 10^-3^	5.43 × 10^-3^	4.62 × 10^-3^	4.11 × 10^-3^	2.62 × 10^-4^	4.12 × 10^-4^	2.88 × 10^-4^	2.09 × 10^-4^
RS3107646	1.82 × 10^-4^	2.83 × 10^-4^	1.37 × 10^-4^	3.20 × 10^-4^	3.97 × 10^-4^	6.11 × 10^-4^	3.03 × 10^-4^	4.60 × 10^-4^
RS2738079	2.68 × 10^-3^	3.60 × 10^-3^	4.27 × 10^-3^	6.79 × 10^-3^	2.58 × 10^-4^	4.08 × 10^-4^	1.34 × 10^-4^	8.47 × 10^-4^
RS6980645	2.49 × 10^-4^	3.82 × 10^-4^	7.71 × 10^-5^	5.05 × 10^-4^	4.00 × 10^-4^	6.15 × 10^-4^	4.10 × 10^-4^	8.70 × 10^-4^

^a^Statistically inappropriate test.

^b^Phenotypic data of un-genotyped individuals were also included.

### Testing association for X-linked SNPs

We performed analysis using GEE adjusted for sex and the two-degrees-of-freedom test, xMQLS. The results of the top ten ranking SNPs using xMQLS are reported in Table 3. The xMQLS gave more significant results compared with other methods (minimum *p*-value = 6.05 × 10^-7^).

**Table 3:** *p*-Values of X-linked SNPs using PLINK allelic association test, GEE adjusted for sex assuming an additive model, and xMQLS, a two-degrees-of freedom test

SNP	PLINK^a^	GEE	xMQLS
RS1025952	1.50 × 10^-3^	1.33 × 10^-4^	6.05 × 10^-7^
RS4557887	1.58 × 10^-3^	1.38 × 10^-4^	6.16 × 10^-7^
RS12688703	1.16 × 10^-3^	1.47 × 10^-4^	8.99 × 10^-7^
RS41345546	8.76 × 10^-4^	9.34 × 10^-4^	4.36 × 10^-6^
RS12010049	4.84 × 10^-3^	3.15 × 10^-4^	7.73 × 10^-6^
RS5913038	1.87 × 10^-3^	5.66 × 10^-4^	9.20 × 10^-6^
RS5913060	2.22 × 10^-3^	5.90 × 10^-4^	9.34 × 10^-6^
RS5912337	2.16 × 10^-3^	5.83 × 10^-4^	9.41 × 10^-6^
RS17003827	2.32 × 10^-3^	6.13 × 10^-4^	9.44 × 10^-6^
RS1021570	2.56 × 10^-3^	6.38 × 10^-4^	1.01 × 10^-5^

^a^Statistically inappropriate test.

## Discussion

The fact that the behavior of the GEE-based methods sometimes deviates from other methods may be explained by the fact that the working correlation matrix has not been specified correctly, especially for nuclear families [9]. This can be a disadvantageous feature of the GEE-based methods for family-based genome-wide association study.

We did not perform simulation studies regarding type 1 error rates of the new methods. However, a good performance of the allelic variants has been reported [4,12], and it is reasonable to expect similar performance from the new tests.

The extended M_QLS _tests can be used for different types of families, and also to incorporate phenotypic information of ungenotyped relatives. Therefore, a better performance can be expected by increasing the number of cases. For this, selecting families with many cases might be more efficient.

The use of an allelic test for X-linked SNPs leads to criticism that males have only half the impact on the analysis as females. Instead, Clayton [7] proposed genotype-based tests for association that treat males as homozygous females. For females, we denote genotypes 0, 1, and 2, and genotypes of males are coded as 0 and 2. Then, X-chromosome specific covariances can be used to calculate genotypic trend tests taking into account the family relationship.

The extended M_QLS _methods are promising. However, these may not be computationally feasible for family-based genome-wide association study. We recommend these tests to be used in a two-stage approach.

## Conclusion

Analyzing family data using all information available in a case-control association study may improve efficiency. Two different subsets of data were considered: one consists of the Offspring Cohort, and the second with nuclear families (Original and Offspring Cohort). To account for relatedness among individuals, we considered first the GEE-based methods. As an alternative, we proposed new methods by extending CA trend test.

To gain efficiency, we also considered the extensions of MQLS test. The last methods utilize most of family information, and therefore might be more efficient than others. Using these methods, we analyzed the real FHS data. The new methods performed well compared with the GEE-based methods.

Adding family information seemed to improve the results. Although only a small number (*n *= 323) was added, the proportion of cases added (20%) was relatively large compared with that in the sibling-only data (6%). And, the gMQLS test might be more efficient because it incorporates all phenotypic information available - even CHD cases of un-genotyped parents.

For X-linked SNPs, equivalent results were obtained: the xMQLS test outperform the GEE-based methods using these specific data. Further work should be done to evaluate the new methods.

## List of abbreviations used

CA: Cochran-Armitage; CHD: Coronary heart disease; FHS: Framingham Heart Study; GEE: Generalized estimating equations; gMQLS: Genotypic test corresponding to the modified quasi-likelihood score; MCA: Modified Cochran-Armitage; M_QLS_: Modified quasi-likelihood score; SNP: Single-nucleotide polymorphism; xMQLS: Two-degrees-of-freedom MQLS.

## Competing interests

The authors declare that they have no competing interests.

## Authors' contributions

H-WU performed the analyses and wrote the manuscript. H-WU and JJH-D participated in the development of the methods, and interpreted the results of the analysis. HJvdW participated in data preprocessing. All authors read and approved the final manuscript.
